# Functional information offers individualized adaptive cancer therapies

**DOI:** 10.18632/oncoscience.607

**Published:** 2024-07-19

**Authors:** R. Craig Herndon

**Affiliations:** ^1^Department of Radiation Oncology, Shannon Cancer Center, Shannon Medical Center, San Angelo, TX 76903, USA

**Keywords:** precision medicine, information theory, biologically guided cancer therapy

The Oxford Computer Science Dictionary contains a general and a technical definition of information [1]. Generally, information is whatever can cause a human mind to change its opinion about the current state of the real world. Technically, information is whatever contributes to a reduction in the uncertainty of the state of a system. Claude Shannon provided an objective measure of information, known as entropy, *H*, by defining it mathematically in terms of the uncertainty associated with the physical process of outputting a message from a source, transmitting it through a channel, and receiving it by an observer in a noisy environment [2]. Following the logic of the technical definition above, information entropy, *H*, increases when there is a reduction in the uncertainty associated with a data set.

The information content in a digital data stream, measured in the on-off elemental form of binary digits (bits) represented by 1’s and 0’s, is the number of bits needed to encode the digital data in the most efficient way [3]. There is a mathematical difference between bits associated with the storage and retrieval of binary data and bits associated with binary information. Bits used for data storage and retrieval are quantified in measurement space where the units of bits have integer-valued digit accuracy, the storage location is filled or not filled [4, 5]. Information is derived from uncertainty space also in the units of bits, however, it has real-valued digit accuracy because optimal encoding of information requires fractional components. An average freeway traffic flow requirement of 2.5 lanes (real-valued) is an abstraction that is accommodated by 3 lanes (integer-valued) in the real world.

Shannon information entropy, based on the outcome probabilities or uncertainties associated with a data stream, has also been defined in terms of signal data uncertainty, an uncertainty often quantified in terms of standard deviation [6]. This statistical connection in turn permits the definition of the information entropy of the signal data, *H* (a physical component defined in terms of absolute uncertainty), as equivalent to the combination of the information of the function that models the signal data, *I_f_* and the information of the uncertainty relative to the signal model, *I_u_*, i.e., *H* = *I_f_* + *I_u_* [5, 7]. Information entropy is a combination of ideal information and uncertainty information. Mathematical models are ideal and can be quantified using functional information, *I_f_*, and thereby placed into a common analytic environment where all model data are in the units of information. Bits are the elemental units for information, however, decimal digits (dits), are used with functional information, *I_f_*, because the mathematical models used by humans are naturally graphed in the decimal format.

The decomposition of Shannon’s information entropy into functional information and uncertainty information allows the analysis of cancer models in the units of information. This creates the opportunity to mathematically connect previously disparate models [5]. Now when the phrase the “two systems are sharing information” is used conversationally, it infers that there is an opportunity to quantify this exchange. For example, the linear-quadratic model (a function of radiation dose), is commonly used to model the cell survival of a tumor subjected to radiation therapy [8]. Measuring tumor cell death requires a biomarker like the quantitative imaging biomarker (QIB) tumor size (a function of time). Functional information opens the pathway to feed data from the QIB model back into the LQ model to individualize treatments [8]. Adaptive changes related to tumor size, determined using the biomarker model, can be used to make dosimetric changes to the bioresponse model.

This application of functional information to adaptive radiation therapy assumes that there is an exchange of information between the bioresponse LQ model and the QIB tumor size biomarker model because the tumor sterilization response to radiotherapy is accounted for by both models. Mathematically, this means there exists a composition of functions that connect the LQ and QIB models [5, 8]. This application of functional information for adapting radiation therapy to individual patients requires an understanding of the systems involved to ensure there is an information exchange between the bioresponse and biomarker model. This adaptive methodology also applies a criterion to the adaptation process where only parametric changes are allowed, i.e., the bioresponse and biomarker models do not change during the adaptation process. This criterion ensures linear changes in bioresponse and biomarker information during the adaptive process, a critical step towards calculation of the adaptive dose [8].

Precision medicine research efforts will benefit from integration of functional information. Since functional information, *I_f_*, is the meaningful component of information entropy, *H*, it transfers the ideal data derived using mathematical models from different domains into the information domain, thus providing a common analytic space [5, 8]. Functional information is an emerging research area that needs participation from all fields of precision medicine.
